# Machine learning model for early prediction of acute kidney injury (AKI) in pediatric critical care

**DOI:** 10.1186/s13054-021-03724-0

**Published:** 2021-08-10

**Authors:** Junzi Dong, Ting Feng, Binod Thapa-Chhetry, Byung Gu Cho, Tunu Shum, David P. Inwald, Christopher J. L. Newth, Vinay U. Vaidya

**Affiliations:** 1 Connected Care and Personal Health Team, Philips Research North America, 222 Jacobs Street, Cambridge, MA 02141 USA; 2 Department of Information Technology, Phoenix Children’s Hospital, Phoenix, AZ USA; 3 Paediatric Intensive Care Unit, Addenbrooke’s Hospital, Cambridge, UK; 4 Department of Anesthesiology and Critical Care Medicine, Children’s Hospital Los Angeles, Los Angeles, CA USA; 5 Department of Pediatrics, Keck School of Medicine, University of Southern California, Los Angeles, CA USA

**Keywords:** Acute kidney injury, AKI, Pediatric critical care, Machine learning, Predictive model

## Abstract

**Background:**

Acute kidney injury (AKI) in pediatric critical care patients is diagnosed using elevated serum creatinine, which occurs only after kidney impairment. There are no treatments other than supportive care for AKI once it has developed, so it is important to identify patients at risk to prevent injury. This study develops a machine learning model to learn pre-disease patterns of physiological measurements and predict pediatric AKI up to 48 h earlier than the currently established diagnostic guidelines.

**Methods:**

EHR data from 16,863 pediatric critical care patients between 1 month to 21 years of age from three independent institutions were used to develop a single machine learning model for early prediction of creatinine-based AKI using intelligently engineered predictors, such as creatinine rate of change, to automatically assess real-time AKI risk. The primary outcome is prediction of moderate to severe AKI (Stage 2/3), and secondary outcomes are prediction of any AKI (Stage 1/2/3) and requirement of renal replacement therapy (RRT). Predictions generate alerts allowing fast assessment and reduction of AKI risk, such as: “patient has 90% risk of developing AKI in the next 48 h” along with contextual information and suggested response such as “patient on aminoglycosides, suggest check level and review dose and indication”.

**Results:**

The model was successful in predicting Stage 2/3 AKI prior to detection by conventional criteria with a median lead-time of 30 h at AUROC of 0.89. The model predicted 70% of subsequent RRT episodes, 58% of Stage 2/3 episodes, and 41% of any AKI episodes. The ratio of false to true alerts of any AKI episodes was approximately one-to-one (PPV 47%). Among patients predicted, 79% received potentially nephrotoxic medication after being identified by the model but before development of AKI.

**Conclusions:**

As the first multi-center validated AKI prediction model for all pediatric critical care patients, the machine learning model described in this study accurately predicts moderate to severe AKI up to 48 h in advance of AKI onset. The model may improve outcome of pediatric AKI by providing early alerting and actionable feedback, potentially preventing or reducing AKI by implementing early measures such as medication adjustment.

**Supplementary Information:**

The online version contains supplementary material available at 10.1186/s13054-021-03724-0.

## Background

AKI affects up to a quarter of pediatric critical care patients [1], and is independently associated with higher mortality, longer lengths of stay, and subsequent development of chronic kidney disease [2–4]. Currently, AKI is diagnosed using Kidney Disease Improving Global Outcomes (KDIGO) clinical practice guidelines, based on serum creatinine and urine output [5]. However, since renal impairment typically precedes increases in creatinine, staging guidelines only detect AKI after renal injury or impairment has already set in. Whilst in pediatric intensive care units (PICU) there are often no specific treatments to reverse AKI after it has developed [6], some studies have shown that early improvements in renal function after AKI may lead to better outcomes [1, 5, 7]. Therefore, early prediction of AKI is important for identifying patients at risk of developing AKI and intervening early to improve outcomes. While AKI is multifactorial in PICU patients, it most commonly occurs following a period of renal hypoperfusion due to hypotension. Simple interventions which might improve renal function include ensuring adequate renal perfusion with intravascular filling or inotropes and avoiding or reducing nephrotoxic drugs. The Acute Dialysis Quality Initiative (ADQI) group recommended developing machine learning models for early prediction of moderate to severe AKI (Stage 2/3) between 48 and 72 h before diagnosis, and suggested that the prediction model should present information about patient measurements contributing to these risks and provide feedback to practitioners regarding potential actionable items [6]. Many research groups have tackled early prediction of AKI using electronic health records (EHR) data [8–11], but no model so far explains the rationale behind specific predictions despite a clear need for explainable and actionable predictions [6, 12]. In pediatric patients where physiology differs greatly with age, developing a predictive model that learns age-appropriate signs of early AKI remains an additional challenge. This study aims to develop a prediction model of AKI for general pediatric critical care patients, running in real-time, that can detect subtle ongoing changes in patient physiology and alert caregivers about patients at high risk of AKI and provide interpretable context and suggested actions. The primary outcome is the ability to predict the onset of moderate to severe AKI 6 to 48 h before it develops. The same model is also assessed on secondary AKI-related outcome measures, including development of any AKI (Stage 1/2/3) and requirement of renal replacement therapy (RRT). To our knowledge, this is the first AKI prediction model built to explain each prediction, and the first multi-center validated model for general pediatric critical care AKI prediction.

## Methods

### Study population

The study cohort included patients from the PICU and cardiothoracic intensive care units (CTICU) of three independent tertiary-care pediatric intensive care centers. The first data set was from a US hospital (Hospital 1) between 2003 to 2011, the second was from a UK hospital (Hospital 2) between 2009 to 2015, and the third was from a US Hospital (Hospital 3) between 2014 to 2019. Records were de-identified for this study, and informed consent was waived as specified in Declerations.

#### Derivation and validation data

Patient data from each of the three centers were split into derivation (70%), validation (15%), and holdout testing (15%) datasets with no patient overlap. One single prediction model was designed and trained using derivation and validation data from all hospitals, and then validated on the holdout test data of each hospital.

#### Cohort extraction

Creatinine measurements were used to label AKI stages using KDIGO serum creatinine criteria [5]. Baseline creatinine was determined by the mean normal creatinine level for age and gender group [14–16]. Urine output criteria were not used due to unreliable records. AKI onset times of patients who developed moderate to severe AKI were labeled as the time of measurement of the first creatinine contributing to AKI Stage 2 or higher; onset times of patients who developed Stage 1 AKI, but not Stage 2/3, were labeled as the time of measurement of the first creatinine contributing to Stage 1; onset times of patients who never developed AKI were selected as a random time during the stay. The following exclusion criteria were applied: (1) patients below one month (neonatal), above 21 years, or without a valid age record, (2) patients with AKI in the first 12 h of ICU stay, (3) patients with length of stay less than 24 h, and (4) if a single patient had multiple encounters, only one encounter was used while remaining encounters were excluded. The included encounter was the one with the highest stage of AKI or the longest length of stay if the highest AKI stage was the same in multiple encounters.

### Outcomes

The primary outcome was prediction of Stage 2/3 AKI during the timeframe 48 to 6 h before onset. For the purpose of training the model on the primary outcome, patients without AKI or those with AKI Stage 1 were labeled as control patients. The trained model was also tested on secondary outcomes, including prediction of any AKI (Stages 1/2/3) and prediction of requirement of RRT.

### Baseline comparator

The renal angina index (RAI), shown to be predictive of AKI at PICU admission [17], was used as a baseline comparator. RAI was calculated prior to AKI onset, as detailed in Additional file 1, and compared to model predictions.

### Predictors

Four types of data elements including vital signs, laboratory values, medication history, and ventilation parameters were extracted from the EHR and used as predictors to train and build the model. Statistics including mean, median, minimum, maximum, change, and last value in the past 30 h were derived for each predictor. For creatinine, an additional creatinine rate of change (CRoC) was determined by calculating the slope (mg/dL/hour) of a line least square fitted to the creatinine measurements within the previous 48 h. Medication data were divided into three categories: low nephrotoxic potential drugs, high nephrotoxic potential drugs, and vasoactive drugs as shown in Additional file 1. The number of times any medication from each category was administered in the previous week was summed and used as predictors. This led to a total of 250 candidate predictors, which were finalized to 15 model input predictors shown in Table 2 in a feature selection process described in Additional file 1.

Predictors were aggregated every six hours to generate AKI risk predictions. After model design and feature selection, the final model was trained on derivation and validation data spanning 48 to 24 h before AKI onset, and tested on holdout data 48 to 6 h before AKI onset. The model is able to make predictions when some predictors are missing in a given timeframe, removing the need for data imputation.

### Statistical analysis methods

**Fig. 1:**
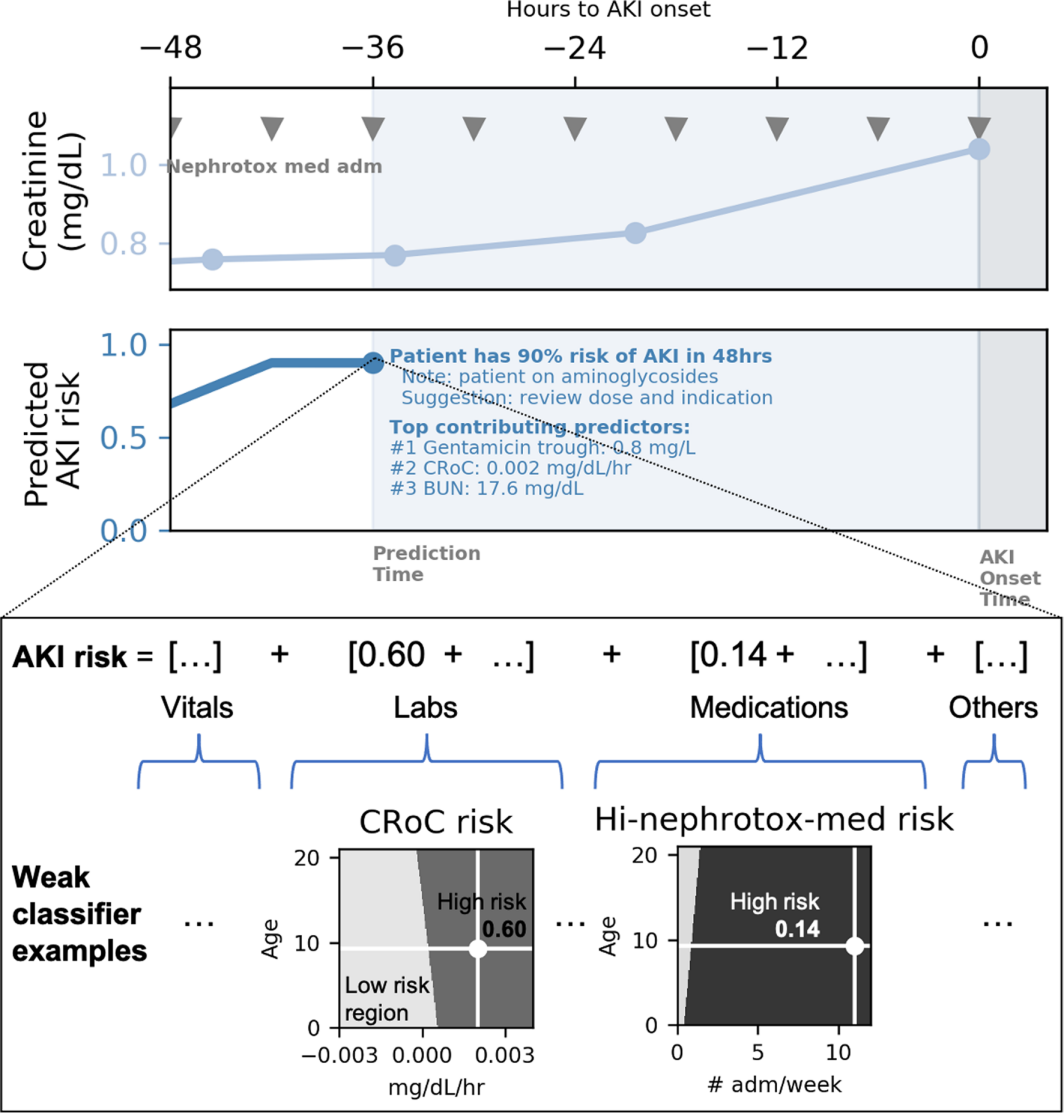
shows an example patient’s AKI disease trajectory and uses the prediction made 36 h before KDIGO Stage 2/3 AKI onset to demonstrate the inner-workings of the model. The top plot shows the patient’s measured serum creatinine values, with AKI onset time referenced as Time 0. The middle plot shows the predicted AKI risk up to prediction time—36 h before AKI onset. It also shows a mockup of the AKI alert that a user would see, including the patient context and suggested actions. The top three predictors contributing the highest risk to this specific prediction are displayed. The bottom portion demonstrates that the model is made up of age-dependent ‘weak classifiers’ of AKI risk based on single predictor values. The predicted AKI risk is the sum of weak classifier risks of all input predictors. Two example weak classifiers are shown. The first is the classifier for creatinine rate of change (CRoC). In the top plot, the example patient’s serum creatinine increases slowly under the AKI threshold prior to prediction time. The increase results in a positive CRoC value and elevated CRoC weak classifier risk of 0.60, as marked on the CRoC classifier plot. At the same time, the patient continuously received drugs with high nephrotoxic potential, shown by triangular ticks marking times of medication administration in the top plot. This results in the high-nephrotoxic drugs classifier risk being elevated to 0.14 (bottom plot). The ellipses (…) in the figure are placeholders for additional predictors not shown due to room constraints

The model was developed following the Transparent Reporting of a Multivariate Prediction Model for Individual Prognosis or Diagnosis guidelines [13]. The derivation and validation data were trained on an age-dependent ensemble machine learning model [15] shown in Fig. 1, which belongs to a class of models that make classifications based on the sum of an ensemble of simpler ‘weak classifiers’. A weak classifier is learned for each predictor and age pair to predict AKI risk given the measured value of that predictor, and can be thought of as a sophisticated lookup table based on the patient’s age and single predictor value, such as age and bilirubin. The model’s consideration of age-dependent risk makes it adaptable to a wide patient age range. Example weak classifiers for CRoC and high nephrotoxic potential drugs are shown in Fig. 1. Since the predicted AKI risk is a sum of all weak classifier predictions, the contribution of each non-age predictor can be separated and ranked for each prediction made. This enables the model to display the top predictors contributing to the highest risk for each prediction, thereby making the AKI risk predictions transparent and interpretable for the user. The predicted risk was adequately calibrated and evaluated by verifying that the calibration curve is close to the diagonal of AKI occurrence rate against predicted risk, as shown in Additional file 1.

## Results

The final cohort demographics including the combined derivation, validation, and holdout cohorts of each hospital are shown in Table 1. The model uses 15 input predictors plus age, as shown in Table 2.

**Table 1:** Cohort demographics of datasets from the three centers

	Hospital 1	Hospital 2	Hospital 3
Cohort size	7329	1220	8314
Age (years)	4 [0.7, 12.1]	2 [0.6, 6.0]	7 [3.8, 14.7]
Female	46%	46%	48%
Length of stay (days)	3 [1, 6]	4 [2, 8]	5 [3, 13]
Mortality	3.5%	3.5%	6.8%
CTICU	28.6%	0%*	12.2%
Any stage AKI	10.9%	19.8%	10.6%
AKI Stage 2 or 3	3.5%	5.7%	5.3%
RRT	0.8%	0.6%	1.7%

Age and length of stay are shown in median [25% percentile, 75% percentile]. *Hospital 2 has no CTICU

**Table 2:** Final predictors categorized by predictor type and ranked by the order in which the predictor was selected by the model

Type	Predictor	Statistic	Unit	*p*-value
Vitals	Shock index*	Max	bpm/mmHg	< 0.001
	SpO_2_	Mean	%	< 0.001
Laboratory values	Blood urea nitrogen	Last	mg/dL	< 0.001
	Serum creatinine rate of change	Last	mg/dL/hr	< 0.001
	Bilirubin	Last	mg/dL	< 0.001
	PaCO_2_	Max	%	< 0.001
	Anion gap	Last	mmol/L	0.005
	White blood cell count (WBC)	Last	10^9^/L	< 0.001
	Serum albumin	Last	g/dL	< 0.001
	Serum chloride	Last	mmol/L	< 0.001
	Gentamicin trough	Last	mg/L	< 0.001
Medications	Number of vasoactive drugs administered	–	–	< 0.001
	Number of high nephrotoxic potential drugs administered	–	–	< 0.001
Ventilation†	Mean airway pressure	Median	cmH_2_O	0.277
Others	Time since admission	–	hours	< 0.001

^*^Shock index = heart rate/blood pressure. †Ventilation-related predictors are treated as missing data for patients not on ventilation. When not available, predictor values are entered as not a number (NAN), which the model is capable of handling

### Performance

**Fig. 2:**
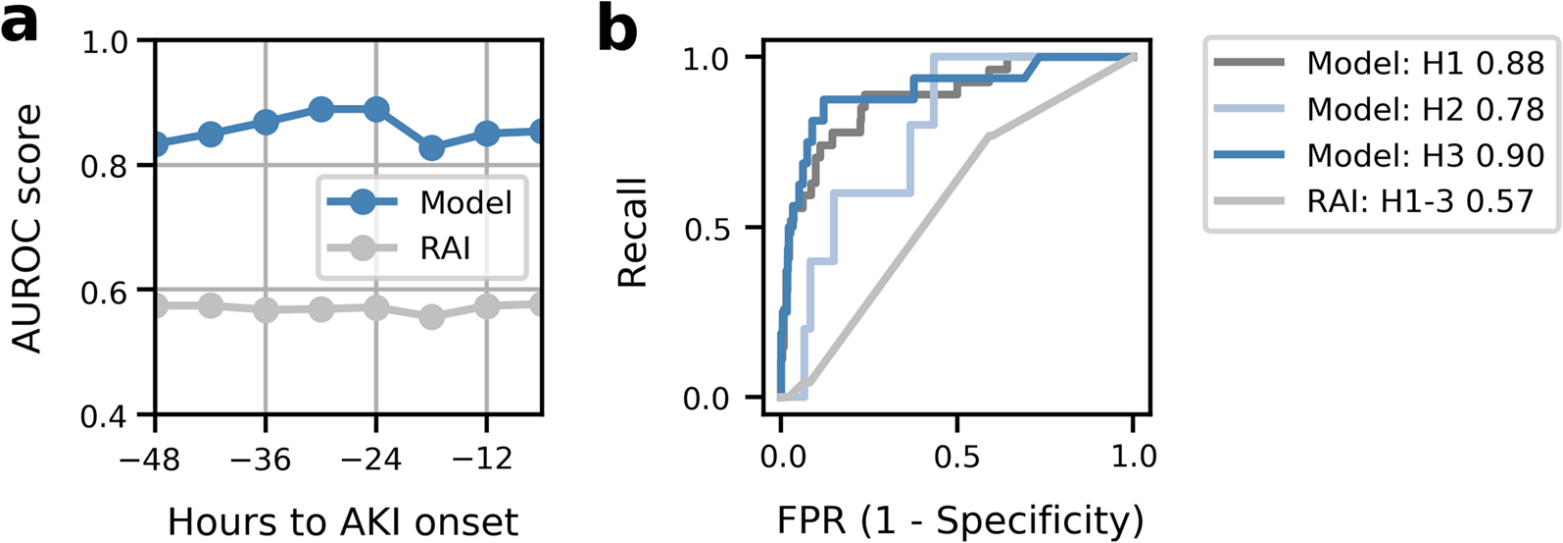
**a** AUROC of the model developed in this study and the renal angina index (RAI). Model AUROC increases closer to onset time, especially inside the training window of 48 to 24 h. **b** The receiver-operator curve at 30 h before onset time for the model and RAI. H1, H2 and H3 represent results from the holdout test data of the three hospitals. RAI results are shown for holdout test data from all three hospitals (H1-3)

The trained model predicts AKI well with AUROC increasing from 0.83 to 0.89 within the training prediction window of 48 to 24 h before AKI onset, decreasing slightly to 0.85 outside the training window, as shown in Fig. 2a. Performance metrics are shown in more detail in Additional file 1. Figure 2b shows that model performance between the two US hospitals (Hospital 1 and 3) were comparable, while the performance was worse on the UK hospital (Hospital 2), likely due to its smaller data size and differences in data characteristics (see Discussion). Performance was slightly better in older patients: in CDC-defined age groups of 1mos to 2 years, 2 to 12 years, 12 to 16 years, and 16 to 21 years, respectively, AUROC were 0.84, 0.90, 0.88, and 0.95. The model showed no performance difference between PICU and CTICU patients.

The trained model can identify 70% of RRT, 58% of AKI Stage 2/3, and 41% of any AKI patients during the prediction timeframe as quantified in Table 3. AKI alerts triggered by the model have a true positive (TP) to false positive (FP) ratio of one-to-one for any AKI, meaning that there was one FP prediction that did not go on to develop any AKI for each TP prediction, which corresponds to a positive predictive ratio (PPV) of 47%.

**Table 3:** Evaluation metrics on the holdout test datasets of all hospitals aggregated across the prediction timeframe for the trained model and renal angina index (RAI)

	Sensitivity		PPV (TP:FP)	
	Any Stage AKI (%)	AKI Stage 2 or 3 (%)	Any stage AKI	AKI Stage 2 or 3
Model	41	58	47% (1:1)	22% (1:4)
RAI	5	3	4% (1:21)	1% (1:98)

Evaluation metrics were calculated from AKI alerts, which were triggered when the model’s predicted AKI risk crossed the pre-determined risk threshold. Sensitivity is computed as the percentage of AKI episodes that were identified by the model among target AKI episodes. Positive predictive value (PPV) is shown alongside true positive (TP) to false positive (FP) ratios for clarity

Moderate to severe AKI patients were identified with a median lead-time of 30 h. 16% of Stage 2/3 patients were identifiable 48 h before onset, increasing to 58% at 6 h to onset. For RRT patients, 70% were identified 6 h or earlier before AKI onset.

The majority of AKI patients predicted by the model received potentially nephrotoxic medications within the prediction timeframe: 40%, 70%, and 79% of TP patients received drugs with high, low, or any nephrotoxic potential within the prediction window, respectively.

### Predictions for example patient

Figure 1 shows predictions for a de-identified patient whose serum creatinine measurements consistently increased but did not exceed KDIGO threshold until AKI onset at Time 0. Assuming the current time is 36 h before onset, bedside practioners have no knowledge of the unknown pending onset of AKI, and continue to give medications with nephrotoxic potential. The middle plot shows what the alert would look like to a caregiver: in addition to noting that the “patient has 90% risk of developing AKI in the next 48 h”, contextual information that the patient is “on aminoglycosides” and which measurements are the main contributors to the high risk are given. Suggested action to “review dose and indication” of medications is also displayed.

### Baseline comparator

In comparison, RAI was less predictive of AKI with AUROC around 0.57 in Fig. 2a and b and sensitivity and specificity shown in Table 3. RAI was calculated without patient stem cell transplantation information, as detailed in Additional file 1.

## Discussion

The machine learning model described here results in a prediction tool for PICU, applicable to patients in a wide age range between 1 month to 21 years, and demonstrates a strong predictive performance up to 48 h in advance of Stage 2/3 AKI onset. The model was trained and validated on multi-center data from three independent PICUs. The model predicts 40% of any AKI episodes, 58% of all Stage 2/3 AKI episodes, and 70% of episodes requiring subsequent RRT, with a ratio of one-to-one false to true alerts relevant to any AKI, as shown in Table 3. The model outperforms a more simple comparator, the RAI, shown to be predictive of AKI in critically ill children [17]. The model predicted onset of Stage 2/3 AKI a median of 30 h before its actual occurrence, thus providing a critical window of time for clinical interventions that might prevent the development of AKI or reduce its severity.

In a consensus paper published four years ago, the ADQI group called for forecasting Stage 2/3 AKI and clinically important AKI-related outcomes for the general critical care population [6]. Notably, ADQI stated that the role of the tool is not only to provide feedback on renal risk, but also to present information about patient measurements contributing to these risks and provide feedback to practitioners regarding potential actionable items [6]. To date, many studies have been published on AKI prediction, but none have focused on building a tool that provides information about the most relevant patient measurements as guidance for action. We built this model with transparency and actionability as main goals, intentionally staying away from ‘black-box’ neural network models that could have achieved better performance. It is also the first multi-center validated AKI prediction model for PICU.

### AKI predictors

This multi-center validated model demonstrates that there exists commonly available EHR data elements, consistent across institutions, that are useful for early-indication of AKI development before creatinine becomes elevated. The 15 predictors used in this model reflect an effort to balance between model transparency and performance: there is a sufficient number of predictors to inform patient state, but not so many to render the model un-interpretable and overfit. Predictors can be considered in three categories: (1) those that causally impact renal health, such as the admistration of nephrotoxic drugs and gentamicin trough values, (2) those that directly reflect renal health, such as CRoC, and (3) those that reflect a more general state of patient health, such as WBC. What is learned about each predictor can be clearly visualized (Fig. 1 weak classifier visualizations) to show what measurement values contribute to low and high AKI risk, allowing users to study the inner-workings of the model before using it in real-time.

### Impacting clinical workflow and improving outcomes

Ultimately, the goal is to use model predictions to intervene early and prevent the development of AKI. The majority (79%) of predicted patients received potentially nephrotoxic medications after predictions were made but before AKI fully developed, which means that these medications could have been stopped, reduced, or replaced by alternate non-nephrotoxic medications where possible, after an early alert. The example patient shown in Fig. 1 exemplifies this: nephrotoxic medications continued to be administered after the patient was identified as high AKI risk by the model. The suggestion made 36 h early to “review dose and indication” of medications could have provided a critical time window towards early recovery.

When deploying predictive models to real-world settings, transparency, interpretability, and actionability are critical in gaining caregiver trust. By presenting not only the predicted risk, but also displaying the top contributing predictors for the alert along with their measured values, and suggested actions, the model allows caregivers to quickly check if the prediction matches their clinical intuition, as shown in Fig. 1. This addresses the need expressed by ADQI for a predictive model to present information about patient measurements contributing to the predicted risks and provide feedback to practitioners regarding potential actionable items [6].

Studies have shown that non-predictive real-time detection of guideline-based AKI reduces the rate of AKI occurrence [18], and we plan to conduct a similar prospective study of prediction-based AKI monitoring to further reduce AKI.

### Performance variability across hospitals

The model performed worse on the UK hospital than US hospitals. This is due in part to an imbalance in data size—the UK hospital has a smaller cohort size compared to other hospitals—so the trained model picks up on pre-AKI data patterns more representative of the other two hospitals. UK hospital patients are younger than those of other hospitals as shown in Table 1. Younger patients were predicted less accurately by the model, so the lower performance could also be due to an over-representation of younger patients. We observed other differences in the data patterns between UK and US hospitals, discussed in more detail in Additional file 1. The dataset from the three hospitals cover different timeframes due to data availability, and should not impact results as management strategies have not evolved.

### Baseline comparator

The baseline comparator RAI, using mainly creatinine to predict AKI [17], performed worse than the model. The better performance of the model is enabled by the use of additional patient information such as medication history and laboratory values. In addition, stem cell transplantation information, originally used in RAI [17] but not in the model, may have contributed to the higher performance of previously reported RAI compared to those computed here. Information fields not readily accessible in the EHR, including patient co-morbidities, diagnostic categories, and contextual information such as stem cell transplantation, were not included given that the goal was to build an all-come PICU model that can automatically assess AKI risk in real-time using only readily available information.

### Prediction frequency

Due to the emphasis of the model on longer-term predictors such as laboratory values, and the disease progression rate of AKI, the model was designed to generate predictions every 6 h. This helps reduce alert fatigue by decreasing the overall possible number of alerts, as clinical decision support systems are recommended to be parsimonious and only alert on the most relevant or severe cases in order to reduce repeated alerts causing alert fatigue [19].

## Limitations

The models uses creatinine alone to stage AKI due to data availability. Up to 20% of AKI patients are diagnosed based on the urine output criteria alone [20], and the lack of urine criteria may have resulted in lower AKI rates than previously described. This impacts the model results in a few ways. First, the model is likely to have lower sensitivity on urine-staged AKI patients and miss more of them. Second, some false positive patients predicted incorrectly to have creatinine-staged AKI may be urine-staged patients, so the reported PPV may increase when urine-staged patients are taken into account. As urine-staged patients have worse outcomes, a future goal is to improve the model when urine data recording is made more reliable.

Though this is the first pediatric AKI prediction model tested on multi-center data, model generalizability is not fully resolved. Given the current retrospective validation results, more validation work needs to be done on larger non-US datasets. The best way to present and integrate the model into the workflow remains to be tested. Performance of the model against admission reason and patient comorbidities is unknown.

## Conclusion

The machine learning model described in this study accurately predicts moderate to severe AKI up to 48 h in advance of AKI onset. The model was validated on three independent centers for general pediatric critical care patients, including PICU and CTICU, and across a wide age range from 1 month to 21 years. The model achieves good performance and may improve outcome of pediatric AKI in clinical settings by providing early alerting and actionable feedback.

## Supplementary Information

**Additional file 1**: AKI_CriticalCare_supplements.docx.

## Data Availability

Datasets were acquired and de-identified at the respective institution before transferred securely to the research site. Data was approved for limited use by the Institutional Review Boards of the respective hospitals, and not publicly available.

## Abbreviations

ADQI: Acute dialysis quality initiative
AKI: Acute kidney injury
AUROC: Area under the receiver-operator curve
EHR: Electronic health records
FP: False positive
KDIGO guideline: Kidney Disease Improving Global Outcomes guideline
PPV: Positive predictive value
RRT: Renal replacement therapy
TP: True positive
WBC: White blood cell count
