# Supplementary material for: Machine learning model for early prediction of acute kidney injury (AKI) in pediatric critical care
Source: Crit Care. 2021 Aug 10;25:288. doi: 10.1186/s13054-021-03724-0 (PMC8353807; doi:10.1186/s13054-021-03724-0)
Supplement: Supplementary file 1 — Additional file 1: AKI_CriticalCare_supplements.docx. [file 13054_2021_3724_MOESM1_ESM.docx]

**Supplementary Material**

**Machine Learning Model for Early Prediction of Acute Kidney Injury (AKI) in Pediatric Critical Care**

Junzi Dong PhD^1^, Ting Feng PhD^1^, Binod Thapa-Chhetry^1^, Byung Gu Cho PhD^1^, Tunu Shum BS^2^, David P. Inwald MB, FRCPCH^3^, Christopher J.L. Newth MD, FRCPC^4,5^, Vinay U. Vaidya MD^2^

**Affiliations:** ^1^Connected Care and Personal Health Team, Philips Research North America, Cambridge, MA; ^2^Department of Information Technology, Phoenix Children’s Hospital, Phoenix, AZ; ^3^Paediatric Intensive Care Unit, Addenbrooke’s Hospital, Cambridge, UK; ^4^Department of Anesthesiology and Critical Care Medicine, Children’s Hospital Los Angeles, Los Angeles, CA; ^5^Department of Pediatrics, Keck School of Medicine, University of Southern California, Los Angeles, CA.

## **Contents**

**eMethods**

- Feature selection
- Renal angina index (RAI) calculation
- eTable 3: age- and gender-based creatinine baseline values
- eTable 4: medications included in each category
- eTable 5: list of candidate predictors not included in final model

**eResults**

- eTable 6. Full prediction performance metrics on primary outcome AKI Stage 2/3 by prediction time
- eTable 7. Full prediction performance metrics on secondary outcome AKI Stage 1/2/3 by prediction time
- eTable 8. Renal angina index (RAI) performance metrics on primary outcome AKI Stage 2/3 by prediction time
- eFigure 1: calibration plot
- Observed data variability across institutions

#### Feature selection

Candidate predictors were selected using the training dataset in a two-step process. In the first step, predictors were filtered to remove co-linearity while keeping those most strongly associated with the AKI label. Among multiple predictors determined to be co-linear (correlation higher than 0.97), only the predictor having highest mutual information with the AKI label was kept. In this step, 250 candidate predictors were selected down to 34 predictors plus age. In the second feature selection step, these 34 predictors plus age were fed to the machine learning model [15], which iteratively selected the next-most informative predictor to maximize performance during the training stage. The final 15 predictors (not including age) selected by the model are shown in Table 2 of the main text. The remaining 19 candidate predictors not used in the final model are shown in eTable 3.

If a predictor was not available in the given timeframe before prediction time, a NAN (not a number) value was extracted instead. The model is able to make predictions when some predictors had NAN values, removing the need for data imputation. Samples with less than two valid numerical predictors were discarded.

#### Renal angina index (RAI) calculation

The renal angina index (RAI) was calculated following the methodology laid out in Fig. 1 of the study by Basu et. al [17], also summarized below. The calculated RAI was the product of an overall risk score component shown in eTable 1 and an kidney injury score component shown in eTable 2. Stem cell transplant information was not available and omitted from the formula, and changes in estimated creatinine clearance (eCCl) was calculated using the most recent serum creatinine and baseline creatinine values. RAI was calculated in an on-going basis similar to how model predictions were made: every six hours from 48 to 6 hours before AKI onset. This allowed RAI and model predictions to be compared side by side.

**Renal angina index (RAI) = Overall risk score × kidney injury score**

#### eTable 1. Overall risk score component of renal angina index (RAI).

| Patient | Overall risk | Overall risk score |
| --- | --- | --- |
| PICU admission | Moderate | 1 |
| Stem cell transplantation | High | 3 |
| Ventilation and inotropy | Very high | 5 |

#### eTable 2. Kidney injury score component of renal angina index (RAI).

| Estimated creatinine clearance (eCCl) | Kidney injury score |
| --- | --- |
| No change | 1 |
| Decrease 0% to 25% | 2 |
| Decrease 25% to 50% | 3 |
| Decrease 50% or more | 4 |

#### eTable 3. Age- and gender-based creatinine baseline values used in KDIGO AKI staging

| **Age** | **Normal Level**  **(mg/dL)** | **Age** | **Normal Level**  **(mg/dL)** |
| --- | --- | --- | --- |
| 2-12 months | 0.2-0.4 | 9-10 years | 0.3-0.7 |
| 1-2 years | 0.2-0.5 | 11-12 years | 0.3-0.9 |
| 3-4 years | 0.3-0.7 | 13-15 years | 0.4-0.9 |
| 5-6 years | 0.3-0.7 | Males 16 yrs+ | 0.6-1.2 |
| 7-8 years | 0.2-0.6 | Females 16 yrs+ | 0.5-1.0 |

#### eTable 4. Three categories of medications used as predictors

| **Medication category** | **Medication list** |
| --- | --- |
| Vasoactive medications | Dopamine  Dobutamine  Epinephrine  Vasopressin  Norepinephrine  Neosynephrine |
| Low nephrotoxic potential medications | Acetazolamide  Amikacin  Chlorothiazide  Furosemide  Ibuprofen  Acyclovir  Captopril  Enalapril  Metolazone  Piperacilin  Scopolamine  Spironolactone  Celecoxib  Indomethacin  Ivig  Ketorolac  Naproxen  Neomycin  Sirolimus  Streptomycin  Valacyclovir  Valganciclovir  Gastrografin  Lithium  Aspirin  Cefotaxime  Ceftazidime  Cefuroxime  Cyclosporine  Dapsone  Foscarnet  Ganciclovir  Lisinopril  Losartan  Pentamidine  Sulfamethoxazole  Voriconazole  Sulfasalazine  Topiramate  Zonisamide  Enalapritat  Ifosfamide  Topiramatezonisamide |
| High nephrotoxic potential medications | Vancomycin  Gentamicin  Tacrolimus  Tobramycin  Amphotericin B  Carboplatin  Cisplatin  Cyclosporin  Cidofovir  Colistimethate  Methotrexate |

#### eTable 5. Candidate predictors after the first feature selection step

| **Predictor type** | **Predictor** | **Temporal** | **Unit** | **Mutual information** | **p-value** |
| --- | --- | --- | --- | --- | --- |
| Vitals | OSI* | Max | - | 0.077 | 0.001 |
|  | etCO2 | Min | mmHg | 0.018 | 0.26 |
|  | Heart rate | Max | bpm | 0.017 | <0.001 |
|  | Temperature | Change | Celsius | 0.007 | <0.001 |
| Laboratory values | BUN to creatinine ratio | Max | - | 0.088 | <0.001 |
|  | MCV | Last | fL | 0.035 | <0.001 |
|  | RBC | Last | million/µL | 0.026 | <0.001 |
|  | Hematocrit | Last | % | 0.023 | 0.119 |
|  | Protein | Last | g/dL | 0.022 | <0.001 |
|  | Vancomycin trough | Last | µg/mL | 0.016 | <0.001 |
|  | Glucose | Change | mg/dL | 0.012 | 0.362 |
|  | Hemoglobin | Last | g/dL | 0.012 | <0.001 |
|  | MCHC | Last | g/dL | 0.011 | <0.001 |
|  | Lactic acid | Change | mg/dL | 0.010 | <0.001 |
| Medication | Number of low nephrotoxic potential drugs administered | Sum | - | 0.023 | <0.001 |
| Ventilation† | PaO2 to FiO2 ratio | Last | mmHg/fraction | 0.035 | 0.479 |
|  | PaO2 to FiO2 ratio | Change | mmHg/fraction | 0.019 | 0.031 |
|  | FiO2 | Last | fraction | 0.017 | <0.001 |
| Others | Weight | - | kg | 0.104 | 0.238 |

**eTable 3.** List of 19 candidate predictors selected in the first but not final feature selection step, ranked by mutual information, which quantifies the amount of information obtained about the AKI label through the predictor value. *OSI: oxygen saturation index = ${100\times FiO_{2}\times MAP}/{SpO_{2}}$. †Ventilation-related predictors treated as missing data for patients not on ventilation.

#### eTable 6. Full prediction performance metrics on primary outcome (AKI Stage 2/3) by prediction time

| **Time to onset** | **AUROC** | **PPV** | **Sensitivity** | **Specificity** | **NPV** |
| --- | --- | --- | --- | --- | --- |
| -48 | 0.83 | 0.16 | 0.34 | 0.94 | 0.98 |
| -42 | 0.85 | 0.17 | 0.37 | 0.94 | 0.98 |
| -36 | 0.87 | 0.18 | 0.41 | 0.94 | 0.98 |
| -30 | 0.89 | 0.22 | 0.56 | 0.94 | 0.99 |
| -24 | 0.89 | 0.23 | 0.61 | 0.94 | 0.99 |
| -18 | 0.83 | 0.20 | 0.50 | 0.94 | 0.98 |
| -12 | 0.85 | 0.25 | 0.57 | 0.94 | 0.99 |
| -6 | 0.85 | 0.28 | 0.56 | 0.95 | 0.98 |

**eTable 4.** Full AKI Stage 2/3 prediction performance metrics by prediction time for validation data from Hospital 1 and Hospitals 2&3, respectively.

#### eTable 7. Full prediction performance metrics on secondary outcome any AKI (Stage 1/2/3) by prediction time

| **Time to onset** | **AUROC** | **PPV** | **Sensitivity** | **Specificity** | **NPV** |
| --- | --- | --- | --- | --- | --- |
| -48 | 0.75 | 0.44 | 0.27 | 0.96 | 0.91 |
| -42 | 0.77 | 0.43 | 0.28 | 0.96 | 0.92 |
| -36 | 0.78 | 0.41 | 0.28 | 0.95 | 0.92 |
| -30 | 0.77 | 0.46 | 0.36 | 0.95 | 0.93 |
| -24 | 0.77 | 0.48 | 0.36 | 0.96 | 0.93 |
| -18 | 0.76 | 0.43 | 0.33 | 0.95 | 0.93 |
| -12 | 0.78 | 0.49 | 0.36 | 0.96 | 0.93 |
| -6 | 0.79 | 0.54 | 0.35 | 0.97 | 0.93 |

#### eTable 8. Renal angina index (RAI) performance metrics on primary outcome (Stage 2/3) by prediction time

| **Time to onset** | **AUROC** | **PPV** | **Sensitivity** | **Specificity** | **NPV** |
| --- | --- | --- | --- | --- | --- |
| -48 | 0.57 | 0.02 | 0.05 | 0.93 | 0.96 |
| -42 | 0.57 | 0.02 | 0.04 | 0.93 | 0.96 |
| -36 | 0.57 | 0.02 | 0.04 | 0.92 | 0.96 |
| -30 | 0.57 | 0.02 | 0.04 | 0.92 | 0.97 |
| -24 | 0.57 | 0.01 | 0.03 | 0.91 | 0.97 |
| -18 | 0.56 | 0.01 | 0.02 | 0.91 | 0.96 |
| -12 | 0.57 | 0.01 | 0.02 | 0.91 | 0.96 |
| -6 | 0.58 | 0.00 | 0.01 | 0.90 | 0.96 |

#### eFigure 1. Calibrated risk on training data


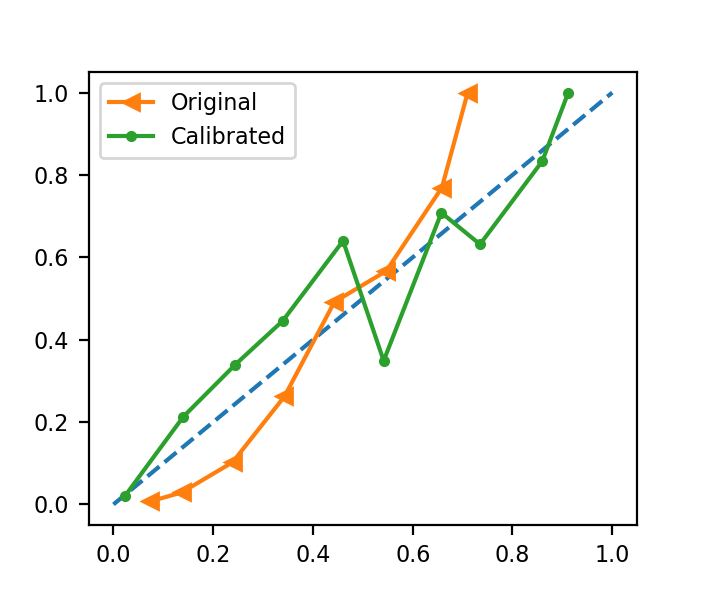


**eFigure 1.** Calibrated risk curve close to diagonal. The rate of AKI occurrence after calibration roughly equates the predicted risk. For example, for patients whose predicted risk is 25%, roughly 25% of patients develop AKI.

#### Observed data variability across institutions

As an example of data pattern variability across UK (Hospital 2) and US hospitals (Hospital 1 and 3), white blood cell count (WBC) laboratory values measured in Hospital 2 have a narrower distribution compared to Hospitals 1 and 3: 56% of measured WBC values from Hospital 2 fall into the normal physiological range between 4.5 to 11 10^9^/L, whereas only 39% and 48% of measured WBC in Hospitals 1 and 3 fall into that range. This means that when using WBC as an indicator of overall patient physiological health and AKI risk, one should have different criteria for Hospital 2 compared to Hospital 1 and 3, which the single model trained on all hospitals cannot capture. The difference in measured WBC may be due to differences in both patient physiology and practice patterns. It is possible that patients in Hospital 2 are healthier and have WBC values in the normal physiological range more frequently. Additionally, differences in practice patterns may also contribute to the difference in measured WBC. It’s possible that WBC is routinely measured in all patients at Hospital 2 but taken more frequently in sicker patients in the other two hospitals, which would explain why the other two hospitals obtained WBC values outside the normal physiological range more often than Hospital 2.

This points to the importance of understanding what pre-disease data patterns are learned by the model and whether these patterns shift when moving to a new institution. At a minimum, one should conduct a thorough comparison of data (predictor) distributions when attempting to implement a pre-trained model in a new institution. Again, the clear visualization or understanding of risk predicted from each type of predictors is critical, as discussed in the main text.
